# Application of Artificial Intelligence in Detecting Dental Anomalies: Current Models, Imaging Modalities, and Future Directions

**DOI:** 10.1002/hsr2.71969

**Published:** 2026-03-02

**Authors:** Mobina Sadat Zarabadi, Zeynab Pirayesh, Shaghayegh Najary, Alireza Jafarzade Ghadimi, Mohammad Behnaz

**Affiliations:** ^1^ USERN Office Qazvin University of Medical Sciences Qazvin Iran; ^2^ Network of Interdisciplinarity in Neonates and Infants (NINI), Universal Scientific Education and Research Network (USERN) Tehran Iran; ^3^ Department of Orthodontics and Dentofacial Orthopedics School of Dentistry Zanjan University of Medical Sciences Zanjan Iran; ^4^ School of Dentistry Shahid Beheshti University of Medical Sciences Tehran Iran; ^5^ USERN Office Shahid Beheshti University of Medical Sciences Tehran Iran; ^6^ Department of Computer Engineering Islamic Azad University Central Tehran Branch Tehran Iran; ^7^ Dental Research Center, Research Institute of Dental Sciences, School of Dentistry Shahid Beheshti University of Medical Sciences Tehran Iran

**Keywords:** anomalies, artificial intelligence, dentistry, machine learning, supernumerary teeth

## Abstract

**Background and Aim:**

As dental anomalies can significantly affect esthetic and function, early detection and diagnosis are crucial for treatment and minimizing potential negative effects. Artificial intelligence (AI) has emerged as a promising tool for the segmentation and detection of dental anomalies in number, morphology, size, position, and structure that may be missed by dentists. This study aimed to investigate the application of various AI models in dental anomaly detection and diagnosis, including supernumerary teeth, tarodontism, impaction, ectopic eruption, and molar‐incisor hypomineralization in both dental radiography and photography.

**Method:**

A comprehensive literature search was conducted in PubMed/Medline, Scopus, Web of Science, and Google Scholar for studies published from the initiate up to 2023 on AI applications in dental anomaly detection. Inclusion criteria encompassed recent AI models utilizing imaging modalities for identifying dental abnormalities, with full‐text availability in English. Studies lacking imaging‐based AI applications or methodological clarity were excluded.

**Results and Conclusion:**

A total of 20 studies assessed various AI models for detecting dental anomalies in radiographic and photographic imaging. Deep learning models, particularly EfficientDet‐D3, nnU‐Net, and ResNeXt, demonstrated the highest accuracy for supernumerary teeth, ectopic eruption, and molar‐incisor hypomineralization, respectively, with most models achieving accuracy rates above 85%. These findings underscore AI's significant potential for automated dental anomaly detection; however, performance varied across different anomalies and imaging modalities, highlighting the need for further optimization. Given the complexity of simultaneous dental anomalies, future research should focus on developing multi‐class AI models capable of detecting multiple conditions concurrently and integrating clinical and radiographic data for improved diagnostic accuracy and treatment planning.

## 1 Introduction

Dental anomalies might be a result of disruptions in the epithelial–mesenchymal interaction during odontogenesis. These disturbances can occur at any stage of tooth development and can impact various aspects, including number, morphology, size, position, and structure [1].

Dental anomalies can remain asymptomatic; however, they can lead to malocclusion and esthetic problems or can cause several complications during dental treatment. Therefore, paraclinical examinations such as radiography should be considered alongside clinical examination [2]. Dental professionals may encounter difficulties distinguishing dental anomalies during clinical examinations [3]. Given the different treatment options required for each anomaly, accurate diagnosis is crucial [4]. Also, for inexperienced clinicians, the diagnosis of dental anomaly is often missed [5]. Thus, an automatic detection tool in radiography can be beneficial for obtaining an accurate diagnosis among clinicians [6].

**FIGURE 1:**
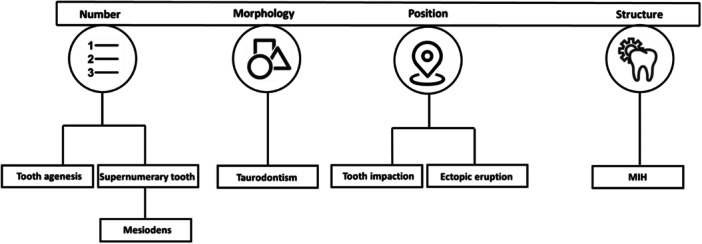
Classification of AI‐based dental anomalies detection in studies.

Nowadays, with the development of artificial intelligence (AI), dental disease diagnosis has been improved [7, 8]. AI plays a crucial role in supporting dentists by enabling swift, data‐driven decision‐making in time‐sensitive situations [9, 10]. Moreover, AI has achieved remarkable results in dental anomaly detection in several studies using convolutional neural networks (CNNs) [6, 11, 12, 13] (Figure 1). Also, it has brought significant results in radiological and pathological diagnosis and enhanced accuracy and precision in abnormality detection [14, 15, 16]. By minimizing human error, it enhances diagnostic accuracy and ensures a consistent standard of care. In addition, it alleviates the cognitive burden on dental professionals [10]. Given the transformative potential of AI in dentistry, dentists must develop a foundational understanding of its concepts and applications to seamlessly integrate this technology into clinical practice and adapt to the evolving healthcare landscape. Herein, this review evaluates the application of AI in detecting specific dental anomalies and explores its clinical implications.

## 2 Methods

### 2.1 Study Design and Search Strategy

This focused review followed an approach to identify and review relevant literature on AI applications in dental anomaly detection. A comprehensive search was conducted in PubMed/Medline, Scopus, and Web of Science databases, supplemented by Google Scholar. The search covered studies published from initiate up to 2023, using predefined keywords, including “artificial intelligence,” “machine learning,” “deep learning,” “dental anomalies,” “dental imaging,” and “radiographic diagnosis.”

### 2.2 Inclusion Criteria

Studies included as follows: relevant English papers with available full text in terms of application of AI models in imaging‐based detection of dental anomalies (e.g., supernumerary teeth, taurodontism, impaction, ectopic eruption, and molar‐incisor hypomineralization) with the use of dental radiography or photography as imaging modalities. Peer‐reviewed research articles, clinical studies, and systematic reviews. Table 2 indicates the results of the included studies.

## 3 A Bit of History

### 3.1 History of AI in Imaging

The integration of AI in imaging has evolved significantly, particularly with the advent of machine learning (ML) and deep learning (DL). While AI was first introduced in the 1950s, its practical application in radiology gained momentum in the early 21st century due to advancements in computational power and data availability [17]. Among DL architectures, CNNs have revolutionized medical imaging, enabling automated feature extraction and pattern recognition beyond human capabilities [18].

Since the introduction of AlexNet in 2012 [19], various CNN models such as VGG [20], ResNet [21], Inception [22], and DenseNet [23] have been adapted for medical imaging, including dental radiology. These models vary in complexity, efficiency, and accuracy, optimizing performance for different imaging tasks [18]. Additionally, object detection frameworks such as Region‐based CNNs (R‐CNNs) and YOLO (You Only Look Once) have enhanced anomaly detection by improving speed and precision in identifying dental abnormalities [24].

### 3.2 History of AI in Dental Imaging

The first attempts at using AI in dental imaging occurred in 1989 when an algorithm for the automatic detection of cephalometry was presented [25]. Later in 2008, a multilayer neural network was designed for detecting proximal caries in extracted teeth, and although the sample size was limited, the study showed promising results when assessed against microscope slides as the gold standard [26]. In 2015, Ronneberge introduced CNNs for dental image segmentation in bitewing radiography [16]. Nowadays, CNNs are considered powerful detection tools, especially in 2D dental radiography [27].

First automatic detection of typical anatomic landmarks was assessed by previous studies, and eventually, the time had come for detection of dental or facial abnormality [28]. A Support Vector Machine (SVM) was used to extract landmarks on cephalometry, and based on typical landmarks, unusual findings were identified as abnormalities in this model with an accuracy of 98% [29]. Certain anomalies, such as tooth impaction, supernumerary teeth, taurodontism, and ectopic eruption, can be detected through 2D (periapical, bitewing, and panoramic) and 3D (Cone Beam Computed Tomography (CBCT)) imaging [30]. Besides the radiographic aspect, many dental anomalies have clinical manifestations, and “photography” is used to capture the clinical appearance of some [31]. However, it should be considered that despite the undeniable accuracy of AI, it must be trained with adequate large datasets. Hence, employing AI with sufficient data will enhance the accuracy and reliability of dental care decision‐making [4]. A summarized timeline of these key milestones in AI development relevant to dental imaging is presented in Table 1.

**TABLE 1:** Milestones in AI development relevant to dental imaging.

Year	Milestone
1989	Automated cephalometric landmark detection
2008	Neural network for proximal caries detection
2012	Introduction of CNNs (AlexNet)
2015	CNN for bitewing radiograph segmentation
2017 onward	Advanced CNNs (VGG, ResNet, DenseNet, and Inception) and object detection frameworks (R‐CNN and YOLO)
Recent years	Integration of AI with CBCT and clinical photography

## 4 Anomalies in Number

### 4.1 Tooth Agenesis

Tooth agenesis is the most prevalent craniofacial abnormality, which is attributed to the absence of one or more tooth buds, and this absence can have either a non‐syndromic or syndromic background, including ectodermal dysplasia and Down syndrome; however, the syndromic type is quite rare. Tooth agenesis can occur in both deciduous and permanent dentition, yet primary teeth are less affected. In permanent dentition, it might be seen commonly in the premaxilla, mandibular, and maxillary premolars with a prevalence ranging from 2.2% to 10.1%, while in primary dentition, maxillary lateral and mandibular central incisors are affected the most [32, 33]. It is associated with the disruption in “tooth formation,” however, the etiology is not fully understood yet. It can be multifactorial and affected by genetic and environmental factors; however, the role of gene mutation is more prominent, and it can be inherited with the autosomal dominant patterns [5]. The early detection of tooth agenesis is rather crucial owing to losing the golden time for following orthodontic treatments. Management of tooth agenesis in adults is complicated due to the possible presence of caries, periodontal conditions, and loss of facial growth potential. Therefore, the sooner and more accurately it is diagnosed, it reduces the burden of possible problems, including esthetics and masticatory functions [33].

### 4.2 AI in Tooth Agenesis

To the best of our knowledge, no focused and specific study has been carried out on AI‐assisted detection of tooth agenesis. However, in several studies, tooth numbering and segmentation were evaluated in panoramic radiography for both permanent and deciduous dentition [34, 35]. In recent studies, AI algorithms were applied to detect the available space for dental implants or small edentulous regions and estimate the missing teeth's position in dental radiography.

Al‐sarem et al. [36] conducted a study about the detection of missing teeth's position based on six different DL models using 500 CBCT in the dataset (Table 2). They aimed to develop various AI models for the assessment of required space for implant insertion. Among all six models, DenseNet169 demonstrated the highest accuracy in both the detection and classification of edentulous regions with an accuracy of 93.3% and 89%, respectively. Park et al. [37] developed DL for the detection of missing teeth's region but this time on panoramic radiography. They developed tooth instance segmentation and missing tooth detection simultaneity and using Mask R‐CNN and Faster R‐CNN, respectively and also, ResNet‐101 was applied as a backbone model. The implemented models in these studies rely on panoramic images, which may be prone to distortions, inconsistencies, or errors that could affect the reliability of AI predictions.

**TABLE 2:** Findings of AI‐based detection system of dental anomalies in studies.

Author	AI models	Accuracy %	Sensitivity %	Precision %	Specificity %	Total dataset	Imaging
**Missing teeth**							
Al‐Sarem et al. 2022 [36]	DenseNet169	93.33	—	94	—	500	CBCT
	MobileNetV3	82.50		85			
	VGG19	85		86			
	ResNet50	90		90			
	VGG16	90		90			
	AlexNet	67.75		77			
Park et al. 2022 [37]	Mask R‐CNN	—	—	92.14*	—	455	Panoramic
	Faster R‐CNN			59.09*			
**Supernumerary teeth (mesiodens)**							
KJ Jeon et al. 2022 [38]	EfficientDet‐D3	99.2	98.3	—	100	600	Periapical
	RetinaNet	98.3	100		96.7		
	YOLOv3	97.5	100		95.0		
Mine et al. 2022 [39]	VGG‐16	82.3	85	nm	79	220	Panoramic
	AlexNet	80.5	82.5		78		
	Inception V3‐TL	80.9	83.3		78		
Ha et al. 2021 [6]	YOLOv3 (internal test)	96.2	95.4	—	96.9	612	Panoramic
	YOLOv3 (external test)	89.8	87.9		91.7		
Ahn et al. 2021 [15]	Inception‐ResNet‐V2	92.4	—	91.6	—	1100	CBCT, Panoramic
	ResNet‐101	92.7		91.1			
	ResNet‐18	91.4		88.3			
	SqueezeNet	83.3		77.9			
Kuwada et al. 2020 [14]	DetectNet	93–96	90–92	100	96–100	550	Panoramic
	AlexNet	80–90	74–84	nm	86–96		
	VGG‐16	52–72	44–74	nm	60–70		
**Taurodontism**							
Duman et al. 2023 [13]	Pytorch U‐Net	—	86.50	78.98	—	434	Panoramic
**Impaction**							
Celik et al. 2022 [40]	YOLOv3	86	—	88	—	440	Panoramic
	RCNN‐ResNet50	79		91*			
	RCNN‐AlexNet	68		86*			
	RCNN‐VGG16	70		87*			
Başaran et al. 2021 [41]	Faster R‐CNN Inception v2	—	96.58	77.93	—	1084	Panoramic
Orhan et al. 2021 [42]	Deep‐CNN (Diagnocat)	86.2	—	—	—	130	CBCT
Kuwada et al. 2020 [14]	DetectNet	93–96	90–92	100	96–100	550	Panoramic
	AlexNet	80–90	74–84	—	86–96		
	VGG‐16	52–72	44–74	—	60–70		
**Ectopic eruption**							
Liu et al. 2022 [11]	Fusion model	—	86	—	90	1580	Panoramic
Zhu et al. 2022 [43]	No‐new‐Net (nnU‐Net)	99	96.7	84.5	99.1	285	Panoramic
	U‐Net	98.2	93.3	75.5	98.4		
	Attention U‐Net	98.1	92.2	74.8	98.4		
	R2U‐Net	98.7	95.4	80.6	98.8		
**Molar‐incisor hypomineralization**							
Schönewolf et al. 2022 [12]	ResNeXt	95.2	78.6	nm	97.3	3241	Intraoral photographs
Alevizakos et al. 2022 [44]	DenseNet121	~92	—	92.86	—	462	Intraoral photographs
	AlexNet	~90		—			
	ResNet34	~90		—			
	ResNet50	~86		—			
	VGG16	~83		83.98			

* Mean average precision (mAP).

While these studies report high accuracy in detecting missing teeth and edentulous regions, they primarily focus on cases where teeth are missing due to extraction or other non‐developmental causes, and there is no way to distinguish them unless knowing the dental history of each patient [36, 37]. Since AI‐based tooth agenesis detection relies solely on dental radiographs or photographs without incorporating genetic or paraclinical data, distinguishing between syndromic and non‐syndromic cases, and should be considered for future research to explore multimodal AI approaches integrating radiographic and genetic data for a more comprehensive diagnosis.

### 4.3 Supernumerary Tooth

Supernumerary teeth are defined as a condition with an extra number of teeth in the dental arch due to splitting of tooth buds. Supernumerary teeth are classified by morphology as conical, tuberculate, supplemental, or odontoma‐related, and by location as mesiodens, paramolars, distomolars, or premolar‐type [45]. They may occur isolated (non‐syndromic) or in syndromic conditions like cleidocranial dysplasia. Potential complications include eruption disturbances, malocclusion, and cystic changes, emphasizing the importance of early radiographic diagnosis and intervention. Although the exact etiology is still unknown factors including genetic or environmental causes may play a role. Supernumerary teeth can be associated with various syndromes, such as cleidocranial dysplasia, Gardner syndrome, and facial deformities, including lip and palate cleft [15, 46]. The prevalence of supernumerary teeth varies between 0.3% and 3.5% for permanent dentition and deciduous dentition, respectively [47]. The most common supernumerary teeth are maxillary central incisors, followed by canines, premolars, and molars with lower prevalence, respectively [48]. The classification of supernumerary teeth is based on their morphology and location. An additional tooth located in the central incisor region is called a “mesiodens.” Odontomas are known as hamartomatous malformations; however, according to Howard's classification, they are categorized as supernumerary teeth [46, 49]. Due to their conical or inverted morphology, supernumerary teeth can be impacted in the dental arch, therefore remaining misdiagnosed and causing delayed eruption or malocclusion, including overcrowding or diastema [39, 47].

### 4.4 AI in Odontoma

AI‐based detection of odontoma has been explored by Okazaki et al. [50] using the AlexNet model across three datasets. Binary classification was applied to determine the presence of anomalies (datasets 1 and 2), while multiclass classification was utilized for identifying the specific type of anomaly (dataset 3). The study demonstrated the feasibility of AI models in detecting and classifying odontomas and supernumerary teeth, suggesting their potential for broader clinical application.

### 4.5 AI in Mesiodens

Panoramic radiography is the most cost‐effective imaging technique for detecting mesiodens, though periapical and CBCT imaging have also been utilized for this purpose [6, 15, 38]. CBCT provides three‐dimensional imaging, which allows precise localization of supernumerary teeth and their relationship with adjacent structures, while periapical radiographs are more detailed and offer better visualization of root morphology, making them useful for assessing individual teeth in detail. Several AI algorithms have been developed for mesiodens detection, with studies showing varying levels of accuracy across different models and imaging modalities.

Mine et al. [39] applied AlexNet, VGG16, and Inception V3‐TL for detecting supernumerary teeth in the premaxilla using panoramic radiographs from children aged 6–9 years in early mixed dentition. VGG‐16 achieved an accuracy of 89%, outperforming the other models. However, in contrast, Kuwada et al. [14] reported lower performance of VGG‐16 in detecting impacted supernumerary teeth in permanent dentition. This discrepancy may stem from differences in dataset characteristics, imaging quality, dentition stage, and model training parameters. Detecting impacted supernumerary teeth presents additional complexity, requiring higher‐resolution imaging and robust feature extraction capabilities, which could influence the model's performance.

Additionally, Ha et al. [6] demonstrated the effectiveness of CNN‐based YOLOv3 in detecting mesiodens across all dentition types in panoramic radiographs while Jeon et al. [38] applied YOLOv3, RetinaNet, and EfficientDet‐D3 to periapical radiographs, with all three models showing promising results. However, AI models still struggle to differentiate between impacted supernumerary teeth and unerupted permanent buds, which remains a significant challenge in clinical implementation [39].

Most studies excluded patients with multiple dental anomalies, limiting the models' ability to classify coexisting conditions. To address this limitation, Okazaki et al. developed a multiclass model capable of classifying supernumerary teeth, odontomas, and standard radiography in a single attempt. This novel approach enhances AI's diagnostic capabilities and highlights the potential for future multi‐class models that can simultaneously detect and classify various dental anomalies [50, 51].

## 5 Anomalies in Morphology

### 5.1 Taurodontism

Taurodontism, caused by the failure in Hertwig's epithelial sheath, mainly involves molar teeth and is detected by intra‐oral radiography (periapical, etc.) and extra‐oral radiography (panoramic and CBCT). An enlarged pulp chamber, apically displaced furcation, and the absence of constriction in the cementoenamel junction (CEJ) are considered a taurodont, and several complications may arise during endodontic treatment of such teeth; for instance, the pulp tissue is easily exposed during cavity preparation of restoration, therefore an accurate detection of this anomaly is rather critical [13, 52].

### 5.2 AI in Taurodontism

According to Duman et al. [13], AI‐based detection systems improve accuracy and reliability of taurodontism diagnosis. Based on the Taurodontism Index (TI) [13, 53]:

$$\frac{\mathrm{height}\;\mathrm{of}\;\mathrm{the}\;\mathrm{pulp}\;\mathrm{chamber}}{\mathrm{length}\;\mathrm{of}\;\mathrm{the}\;\mathrm{roof}\;\mathrm{of}\;\mathrm{the}\;\mathrm{pulp}\;\mathrm{chamber}\;\mathrm{to}\;\mathrm{root}\;\mathrm{apex}}\times 100=\;\text{TI}$$

A U‐Net model for panoramic radiographs was shown to be highly sensitive and precise when identifying taurodontism. A major limitation of current AI models is that they are primarily based on binary classification, which limits their ability to detect coexisting anomalies such as microdontia, dens invaginatus, and amelogenesis imperfecta, or to identify syndromic associations such as ectodermal dysplasia and Down syndrome. Furthermore, the inter‐observer variability in assessing taurodontism highlights the need for AI models that are more consistent and reproducible among human experts. Future AI research should focus on developing multiclass classification models that can detect multiple dental anomalies simultaneously and incorporate syndromic associations for a more comprehensive diagnostic approach [13].

## 6 Anomalies in Position

### 6.1 Tooth Impaction

Tooth impaction is among the most common dental conditions indicated by failure of eruption and mostly involves third molars [40, 42]. When half to three‐quarters of the final root length is completed, it usually erupts; however, various causes can lead to delayed eruption or impaction. Impacted teeth are mostly asymptomatic and are diagnosed when they have caused various complications and require multidisciplinary approaches [54]. Due to the conical and inverted morphology of supernumerary teeth, they might remain unerupted, therefore supernumerary teeth and impaction might occur simultaneously [39].

### 6.2 AI in Tooth Impaction

As mentioned before, AI models, including AlexNet and VGG16, were successfully applied to detect impacted supernumerary teeth located in the premaxilla in both permanent and primary dentitions [14, 39]. However, unlike Kuwada et al. [14], Mine et al. [39] used a more complex dataset of mixed dentition to detect impacted teeth. Both studies used datasets from a single institution and no multicenter validation was performed. Additionally, Mine et al. [39] omitted challenging data that was difficult for clinicians to diagnose, which could result in biased model performance. Also, accurate detection of the impacted third mandibular molar is now provided by the deep‐CNN system in the study of Orhan et al. [42], and besides the impaction detection, the proximity of anatomical structures such as the inferior alveolar nerve or maxillary sinus was assessed additionally. Celik [40] conducted a study where one‐stage technique with YOLOv3 and two‐stage techniques with Faster RCNN with backbones were developed for third molar impaction and YOLOv3 outperformed Faster RCNN with different backbones, including ResNet, AlexNet, and VGG16, in terms of mAP and accuracy, which may be related to the Multi‐label classification of this model. However, the dataset used to train the models was primarily focused on mandibular third molars, given their wider prevalence. Expanding and including diverse cases may result in obtaining more robust and reliable results.

### 6.3 Ectopic Eruption

Abnormal positioning of tooth eruption leads to ectopic eruption (EE) and permanent first molars are mainly involved when erupting under the distal margin of the second primary molars. EE has brought multiple complications, including resorption in the distal margin of second primary molars and the following early loss, reduction of interdental space, premolars impaction, and malocclusion; thus, orthodontic or extraction approaches are required, and it is noteworthy that early diagnosis and treatments can ensure the proper responses [11, 43, 55].

If a 7‐year‐old child's first permanent molar does not erupt, it may indicate the possibility of EE, and additional radiographic evaluations must be conducted. Panoramic radiography is the gold standard for the diagnosis of EE, which relies on the experience of evaluators and can lead to misdiagnosis, therefore automatic detection seems to be beneficial [11].

### 6.4 AI in Ectopic Eruption

According to the articles, AI showed different results in EE detection. Zhu et al. [43] concluded that nnU‐Net (no‐new‐Net) outperformed three pediatric dentists in detection and segmentation of EE on panoramic radiographs in terms of precision, accuracy, f1 score, and mIoU. The reported intraclass correlation coefficient (ICC) between dentists was 0.77, which may reflect the inherent challenges of diagnosing EE from panoramic radiographs due to image distortion and overlapping structures. To address the limitations of panoramics, which challenge even experienced clinicians, additional imaging modalities, such as CBCT, can be incorporated. Integrating labels from CBCT alongside panoramic radiographs could enable AI models to handle more complex cases, enhancing their ability to detect ectopic eruption in both 2D and 3D images, ultimately leading to more reliable and precise results. This can aid clinicians by providing more accurate diagnoses and improving treatment planning. nnU‐Net, a variant of U‐Net, performs based on selecting the best model effect according to the characteristics of each dataset by adjusting various parameters, such as data pre‐processing and training methods [43]. This adaptability allows nnU‐Net to enhance model effectiveness when encountering new datasets, saving time on parameter selection and experimental tuning. In spite of the study's strong performance, one major limitation is the small dataset size, since EE is a rare event. A small sample size may limit the generalizability of the model, making it difficult to perform consistently across various patient populations. The integration of AI models like nnU‐Net into clinical practice holds potential, but it is crucial to consider factors such as dataset size, model generalizability, and adaptability to ensure consistent, reliable performance in real‐world settings. Liu et al. [11] had a different point of view. They concluded that despite the high speed of AI‐based models, they are not accurate enough to detect EE compared to manual detection methods [11]. In comparison to the aforementioned studies, Liu et al. used more panoramic radiography (2960 regions), while Zhu et al. benefited from 438 regions in the dataset [11, 43]. Larger datasets may provide a more robust AI model since they provide a wider range of cases for training, which can enhance generalizability and accuracy, whereas smaller datasets may result in overfitting and reduced reliability.

## 7 Anomalies in Structure

### 7.1 Molar‐Incisor Hypomineralization

Molar‐incisor hypomineralization or MIH is a developmental condition that occurs owing to diminished activity of ameloblasts and disturbance in enamel secretion and maturation [56]. In clinical evaluations, it is indicated by the hypomineralized white to brownish‐yellow enamel defects in the shape of pits and grooves located in the buccal or lingual side of the first permanent molar or incisor [57, 58]. MIH teeth are more prone to caries due to the weak structure of the enamel and early diagnosis is quite important owing to accelerate remineralization treatment [44, 58]. The accurate diagnosis of MIH is challenging due to the various clinical appearances, and it can be misdiagnosed by conditions such as white spot lesions and amelogenesis imperfecta [44].

### 7.2 AI in MIH

MIH is diagnosed more by clinical examinations than radiography; therefore, AI models were used along with photography imaging. In the study of Alevizakos et al. [44], various CNN models were designed to detect and classify MIH in frontal incisor photography, amelogenesis imperfecta, fluorosis, and white spot lesions. DenseNet121 performed better than VGG16 with precision of 92.86% over 83.98%, which may be related to its structure with densely connected layers that facilitate feature propagation. Schönewolf et al. [12] used molar photography as well, however despite the other study mentioned above, pictures including other developmental anomalies, including amelogenesis imperfecta, were excluded. However, DenseNet121 used by Alevizakos et al. was more effective for classifying MIH among multiple dental conditions, whereas the CNN in Schönewolf et al. performed well only in distinguishing MIH from normal teeth but faced challenges accurately detecting MIH due to limited data and the wide variation in MIH appearance.

Despite promising results, clinical challenges still remain; limited and imbalanced datasets resulted from the rare nature of the condition can lead to overfitting of the model and enhancing challenges in detecting certain categories due to the underrepresentation of clinical cases, respectively [44]. The variation in the image quality can affect the model's performance and requires extensive image preprocessing to ensure uniformity. So pictures that are not taken correctly, such as improper exposure or contamination with saliva, must be excluded [12]. MIH presents a variety of clinical manifestations, which makes it hard to differentiate from other similar pathologies and variation in accuracy of the AI model [44]. For the model to perform well across different populations the dataset should cover international variability in terms of skin tones, enamel discoloration, and image quality which was not addressed in the study by Alevizakos et al. [44]. In future studies adopting multimodal approaches, such as combining clinical photography with radiographic imaging, may be beneficial. The integration of diverse imaging modalities could enhance AI diagnostic accuracy and overcome current limitations associated with the variability of clinical presentation and image quality, which can lead to improved early and precise MIH detection in clinical settings.

## 8 Future Direction

Currently, many AI studies focus on detection of one anomaly at a time and often exclude patients with coexisting conditions, which limits their clinical applicability. To advance AI‐based dental anomaly detection, future research should focus on developing multi‐class classification models that are able to identify multiple and similar anomalies simultaneously, this can lead to mirroring real clinical complexity and improving diagnostic efficiency.

Integrating and combining clinical data, and different imaging modalities to develop the prediction model is a promising avenue to enhance diagnostic accuracy and can also serve clinicians as a diagnostic aid in challenging cases. Labeling based on clinical examination and clinical photographs provides details of soft tissues and surface anatomy, while radiographs (such as panoramic X‐rays or CBCT scans) reveal underlying bone and tooth structure. Merging these sources can give a holistic view of a patient's condition that allows AI to correlate findings across modalities.

AI models for imaging tasks are computationally intensive and often require significant processing power and memory, additionally large and diverse multicenter datasets are needed to obtain generalizable results. Improvements in availability of high‐performance GPUs allow for training more complex models on high quality images and larger datasets and enable quicker inference times which can expand the potential for AI applications in dentistry.

Many published models have been developed on single‐center data with no external validation, this can introduce bias and limit generalizability. For multi‐center data sharing, secure, standardized frameworks with considering appropriate anonymization and patient consent should be established. In the long run, such collaboration would produce AI models that are trained and tested on globally diverse patient data, making them more robust across different populations and clinical settings while upholding patient privacy and data security.

## 9 Conclusion

Multiple AI models have demonstrated promising results in terms of enhancing diagnostic accuracy and clinical efficiency across various dental anomalies, including ectopic eruption, impacted teeth, and molar‐incisor hypomineralization. However, the clinical application of AI faces some practical and ethical challenges, such as limited dataset sizes, variability in data quality, concerns regarding patient data privacy, and model generalizability across diverse populations. Ethical concerns must be addressed by developing clear ethical frameworks and practical guidelines. AI can aid clinicians as a supplementary tool by providing sophisticated, data‐driven insights that enhance treatment planning and patient care. Future research should prioritize employing larger, more diverse datasets to improve model robustness and accuracy. Moreover, multimodal diagnostic approaches such as combining clinical and radiographic imaging, or integrating different imaging modalities will be valuable. Lastly, the advancements in this field may help AI become a reliable, integrated partner in clinical dentistry, improving both diagnostic precision and patient outcomes of rare dental conditions.

## Author Contributions

**Mobina Sadat Zarabadi:** conceptualization, data curation, formal analysis, methodology, investigation, writing – original draft. **Zeynab Pirayesh:** methodology, software, data curation, formal analysis, writing – review and editing, investigation. **Shaghayegh Najary:** methodology, investigation, validation, visualization, writing – review and editing. **Alireza Jafarzade Ghadimi:** methodology, software, data curation. **Mohammad Behnaz:** conceptualization, methodology, supervision, validation, writing – review and editing.

## Funding

The authors received no specific funding for this work.

## Disclosure

The lead author Mohammad Behnaz affirms that this manuscript is an honest, accurate, and transparent account of the study being reported; that no important aspects of the study have been omitted; and that any discrepancies from the study as planned (and, if relevant, registered) have been explained.

## Conflicts of Interest

The authors declare no conflicts of interest.

## Data Availability

The data that support the findings of this study are available from the corresponding author upon reasonable request.
